# High-Throughput Human Complement C3 N-Glycoprofiling Identifies Markers of Early Onset Type 1 Diabetes Mellitus in Children

**DOI:** 10.1016/j.mcpro.2022.100407

**Published:** 2022-08-27

**Authors:** Dinko Šoić, Toma Keser, Jerko Štambuk, Domagoj Kifer, Flemming Pociot, Gordan Lauc, Grant Morahan, Mislav Novokmet, Olga Gornik

**Affiliations:** 1 Faculty of Pharmacy and Biochemistry, University of Zagreb, Zagreb, Croatia; 2 Genos Glycoscience Research Laboratory, Zagreb, Croatia; 3 Department of Clinical Research, Steno Diabetes Center Copenhagen, Herlev, Denmark; 4 Faculty of Health and Medical Sciences, University of Copenhagen, Copenhagen, Denmark; 5 Centre for Diabetes Research, Harry Perkins Institute of Medical Research, Perth, Western Australia, Australia; 6 University of Melbourne, Parkville, Victoria, Australia

**Keywords:** C3 protein, glycopeptides, LC-MS, N-glycosylation, type 1 diabetes onset, Con A, Concanavalin A, HILIC, Hydrophilic interaction chromatography, MBL, Mannose-binding lectin, T1D, Type 1 diabetes

## Abstract

Recently, it was shown that children at the onset of type 1 diabetes (T1D) have a higher proportion of oligomannose glycans in their total plasma protein N-glycome compared to their healthy siblings. The most abundant complement component, glycoprotein C3, contains two N-glycosylation sites occupied exclusively by this type of glycans. Furthermore, complement system, as well as C3, was previously associated with T1D. It is also known that changes in glycosylation can modulate inflammatory responses, so our aim was to characterize the glycosylation profile of C3 in T1D. For this purpose, we developed a novel high-throughput workflow for human C3 concanavalin A lectin affinity enrichment and subsequent LC-MS glycopeptide analysis which enables protein-specific N-glycosylation profiling. From the Danish Childhood Diabetes Register, plasma samples of 61 children/adolescents newly diagnosed with T1D and 84 of their unaffected siblings were C3 N-glycoprofiled. Significant changes of C3 N-glycan profiles were found. T1D was associated with an increase in the proportion of unprocessed glycan structures with more mannose units. A regression model including C3 N-glycans showed notable discriminative power between children with early onset T1D and their healthy siblings with area under curve of 0.879. This study confirmed our previous findings of plasma high-mannose glycan changes in a cohort of recent onset T1D cases, suggesting the involvement of C3 N-glycome in T1D development. Our C3 glycan-based discriminative model could be valuable in assessment of T1D risk in children.

## Highlights

• Newly developed method for C3 enrichment and N-glycoprofiling from human plasma.
• Site-specific C3 N-glycan profile obtained from 10 μl of blood plasma.
• C3 N-glycan profile is stable in a healthy individual.
• Significant changes of C3 N-glycosylation accompanying the onset of type 1 diabetes.
• C3 N-glycome profile could be a novel risk factor in type 1 diabetes onset.

## Graphical Abstract

**Figure:**
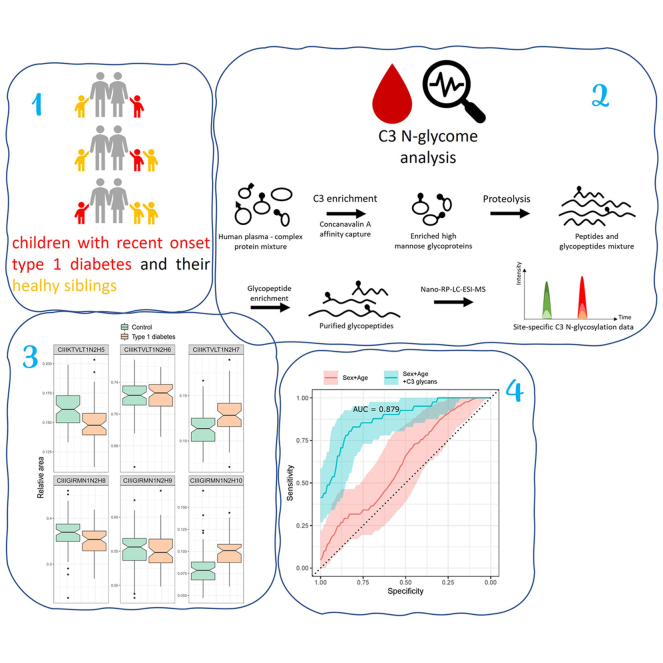

## In Brief

Changes in glycosylation are known to modulate inflammatory responses, so glycans are more and more being considered as biomarkers of various diseases. Little is known about complement component C3 N-glycome as proper methods for its analysis are lacking, so we developed a novel high-throughput LC-MS method for C3 plasma enrichment and subsequent glycome analysis. We demonstrated that C3 N-glycan profile is stable in a healthy individual, and results showed that C3 N-glycosylation profile is significantly changed in type 1 diabetes.

Over the past decades, there has been increasing evidence suggesting a role of the complement system in type 1 diabetes (T1D). This evidence includes genetic studies showing association of allelic variants of complement proteins with autoimmune diabetes; ability of islet cell antibodies to fix complement on the surface of beta pancreatic cells promoting their lysis; and animal and human studies confirming activation of the complement system early in disease pathogenesis (1, 2, 3, 4). Also, several studies have found that serum concentrations of mannose-binding lectin (MBL) were significantly elevated in subjects with T1D (3, 5). MBL, an indicator of the lectin complement pathway, strongly binds to repetitive carbohydrate structural patterns mainly found on pathogen surfaces consisting of mannose, N-acetyl-d-glucosamine, glucose, and fucose but shows no affinity for binding to galactose and the terminal sialic acid of oligosaccharide chains present on the host–cell surface (6). Increased levels of MBL in combination with uncontrolled hyperglycemia may thus result in increased complement activation and contribute to and sustain local chronic inflammation and tissue damage (3, 7). Studies on N-glycosylation of plasma proteins in T1D also suggested an interplay of MBL and glycosylation in the etiology of the disease (8).

N-glycosylation is a ubiquitous cotranslational and posttranslational modification that enriches protein structure and function; it is indirectly encoded by the genome and considerably influenced by environmental factors (9, 10, 11). Changes in N-glycosylation have been described in different diseases including T1D and are increasingly considered as biomarkers of ongoing pathological conditions. Previous studies demonstrated that the N-glycan profile of total serum proteins is altered in adult T1D patients with kidney disease and that N-glycosylation is associated with T1D complications (12, 13). Recently, we demonstrated that total plasma N-glycans were changed in children with early onset T1D and that they show great value [with a high area under receiver-operator curve (AUC) of 0.915] in discriminating between children with T1D and their healthy siblings (8).

Among the observed associations in that study were high-mannose N-glycan levels. Considering the role of complement system in T1D development and the fact that C3 complement component is a protein occupied with these structures, we hypothesized that C3 could be the source of the reported total plasma N-glycan changes of high-mannose levels in T1D early onset. Therefore, we performed a C3 N-glycome analysis in order to possibly reveal a novel risk factor contributing to T1D development.

As the most abundant complement protein with plasma concentrations of around 1.2 mg/ml, C3 has a distinctly resourceful role in immune surveillance. As an intersection of different activation pathways, it inflates the complement response, employs direct effector functions, and helps to coordinate downstream immune responses (14). This unique immune mediator is a 185 kDa glycoprotein encoded by the *C3* gene on chromosome 19 and consists of a singly disulfide linked β (75 kDa) and α (110 kDa) chains. Though C3 contains three potential N-linked glycosylation sites, only two are occupied since Asn 1595 on the α-chain has been shown not to be glycosylated (14, 15). Interestingly, C3 is unusual among hepatocyte-derived glycoproteins containing only high-mannose N-glycans. Glycan analysis of separated C3α and C3β subunits revealed that Man_8/9_GlcNAc_2_ are located on the α subunit, while the β subunit contains smaller Man_5/6/7_GlcNAc_2_ N-glycans (16, 17, 18). The presence of a monoglucosylated oligomannose N-linked glycan Glc_1_Man_9_GlcNAc_2_ was also reported, being the first finding of monoglucosylated glycan on a human serum glycoprotein from nondiseased individuals (18).

Different animal studies indicated that complement component C3 plays a direct role in streptozotocin-induced autoimmune diabetes and that myeloid-derived suppressor cells play a role in resistance to diabetes in absence of C3 (1, 4). The presence of activated C3 in glomeruli and glomerular capillaries of animal models also supports its role in diabetic nephropathy (19, 20). Moreover, a longitudinal study analysing 15 complement polymorphisms reported that the rs2230199 variant of *C3* showed a significant association with clinical T1D among HLA-DR4/4 carriers (2, 4).

Although the basic characterization of complement C3 N-linked glycans has already been done, the workflows used for C3 isolation from human plasma were mostly preparative and not suitable for studies of smaller volumes of samples nor for high throughput applications. Moreover, N-glycan analyses were mainly done using glycan standards and high-performance liquid chromatography on released glycans, so that site-specific information is lost, and contaminating glycoproteins make the results more difficult to interpret (21, 22, 23). Finally, these methods were not mass spectrometry compatible and could therefore not be used for glycopeptide analysis.

Here, we present a novel cost-effective glycoproteomic workflow for a high-throughput and site-specific N-glycosylation LC-MS analysis of human C3 on a glycopeptide level, as well as evaluation of its potential for differentiating recent onset T1D in children. The high-throughput LC-MS glycopeptide approach guarantees that the changes seen originate from C3 protein and facilitates the value of C3 N-glycoprofiling in T1D risk assessment.

## Experimental Procedures

### Experimental Design and Statistical Rationale

The method was developed and optimized using human plasma standard made from pooled plasma of the population for the intraindividual temporal stability study described below. Repeatability of the method was determined by measuring a coefficient of variation (CV) of the pooled plasma standard technical replicates. The workflow was validated for intraday and interday precision for all the glycoforms analyzed. All glycoforms identified and manually annotated from the pooled plasma standard were included for data analysis in the intraindividual temporal stability study and the T1D study. Extracted signals were summed and normalized to total integrated area per glycosylation site in order to remove variation in signal intensity between samples, allowing for their comparison.

The intraindividual temporal stability study was performed to evaluate the C3 N-glycome stability within a healthy individual under physiological conditions. Samples from 14 healthy and age-matched male individuals were measured at three time points: at the beginning of the study and after 6 and 10 weeks. Before the analysis, all samples were randomized across a 96-well plate using Microsoft Excel 2016 software (Microsoft Corp) and then analyzed.

A total number of 145 participants were included in the type 1 diabetes study: 61 children and adolescents (1–16 years) newly diagnosed with T1D and their 84 (4–22 years) unaffected siblings. Each participant was sampled once, and all samples were randomized across two 96-well plates before the analysis.

### Participants in the Intraindividual Temporal Stability Study

Fourteen healthy male individuals (age 19 ± 0.7 years) participated in the study. All participants were screened for cardiovascular diseases, muscle injuries, or ongoing medical treatment before their inclusion into the experimental protocol. Participants were instructed to refrain from alcohol and cigarette consumption as well as antioxidant supplementation throughout the study. The blood samples were taken from each participant at three time points: at inclusion, after 6 weeks, and after 10 weeks. The blood was collected in vacuum tubes containing EDTA with 20-G straight needle venipuncture from the antecubital vein. The EDTA tubes were immediately centrifuged at 1370*g* for 10 min to separate erythrocytes from plasma. Subsequently, plasma supernatant was aspirated into a series of 1 ml aliquots and stored at −80 °C until analysis.

### Participants in the Type 1 Diabetes Mellitus Study

Plasma samples of 61 newly diagnosed children and adolescents (median age of 10, age range 1–16), as well as 84 unaffected siblings (median age of 11, age range 4–22) derived from the Danish Registry of Childhood and Adolescent Diabetes (DanDiabKids) (24) were included in this study. The Registry contains samples from T1D patients collected within 3 months of the disease diagnosis, as well as samples of their healthy siblings. Unaffected siblings were included in the study if a sample from their biological sibling with T1D was available in the registry. Per some affected individuals, multiple siblings were included in the study (from 1 to 4 per affected individual), but for most of the cases, sample of only one unaffected sibling was available. Year of sampling for unaffected siblings ranged from 1997 to 2000, and the last registry data extraction and disease status check for unaffected siblings was performed in January 2019. In total, 145 subjects from 61 families participated in the study, and their demographic data are summarized in supplemental Table S1.

### Ethics Statement

Both studies are designed in accordance with the Declaration of Helsinki and supported by signed informed consent from all the participants, their parents, or guardians. The study was approved by the ethics committee of the University of Zagreb, Faculty of Pharmacy and Biochemistry and the Danish Ethical Committee, respectively.

### Enrichment of C3 From Human Plasma Using Concanavalin A Lectin Affinity Matrix

Concanavalin A (Con A)-Sepharose 4B (Global Life Sciences Solutions) resin slurry (10 μl) was placed into 96-well polypropylene filter plate (Orochem Technologies Inc) to provide a 1:1 ratio of slurry to sample volume and preconditioned thrice with a binding buffer (20 mM Tris, pH 7.4, 0.5 M NaCl, 1 mM CaCl_2_, MgCl_2_, MnCl_2_). Plasma samples (10 μl) were diluted with 90 μl of binding buffer, loaded onto the conditioned resin, and incubated overnight at 4 °C while shaking (25). After washing the lectin matrix thrice with 200 μl of binding buffer, glycoproteins were eluted from the lectin media with 100 μl of elution buffer twice [200 mM methyl α-D-mannopyranoside (Sigma-Aldrich)] in 0.1 M acetic acid [(Merck KgaA), pH 3.0]. The washing was performed on a vacuum manifold (Pall Corp), and the elution was done by 5-min low-speed centrifugation. The eluates were immediately dried down in SpeedVac Vacuum Concentrator (Thermo Fisher Scientific) until digestion.

### Glycoprotein Denaturation and Glu-C Digestion

Dried enriched samples were resuspended and denaturated by incubation with 48 μl of 15% (v/v) 2-propanol (Merck KgaA) in 0.1 M ammonium bicarbonate (Acros Organics) at 60 °C for 10 min (26). For digestion, 2 μl of 0.5 U/μl sequencing grade endoproteinase Glu-C (Sigma-Aldrich) was added in a 1:50 protease:protein ratio (w/w), and the samples were incubated for 18 h at 37 °C in a foil-sealed plate.

### Hydrophilic Interaction Chromatography–Based Solid-Phase Extraction Glycopeptide Enrichment

Glycopeptide hydrophilic interaction chromatography (HILIC)–based solid-phase extraction enrichment following Glu-C digestion was done using Chromabond HILIC silica beads (Macherey-Nagel GmbH & Co) (27). A 50 mg/ml suspension of HILIC beads was first prepared in 0.1% (v/v) TFA (Sigma-Aldrich) on a magnetic stirrer, and 100 μl of suspension was added to each well of the Orochem OF1100 filter plate (Orochem Technologies Inc). Solvent was removed using vacuum manifold (Pall Corp). All wells were prewashed twice with 250 μl of 0.1% TFA (v/v) and equilibrated two times with 250 μl of 0.1% TFA in 90% acetonitrile (ACN) (v/v) (VWR International). The samples were diluted with 450 μl 0.1% TFA in ACN to a final concentration of 90% ACN and applied to the preconditioned beads. Unbound impurities were washed with 250 μl of 0.1% TFA in 90% ACN twice. Enriched glycopeptides were eluted with 200 μl 0.1% TFA into a clean skirted PCR plate by low-speed centrifugation. The eluates were immediately dried down in a SpeedVac Vacuum Concentrator (Thermo Fisher Scientific) and stored at −20 °C until analysis.

### Nano-LC-ESI-MS(/MS) Analysis of Purified Glycopeptides

Analysis of glycopeptides was performed on a ACQUITY UPLC M class nano-LC system (Waters) coupled to Compact Q-TOF mass spectrometer (Bruker Daltonik GmbH). The nano-LC was coupled to the MS using CaptiveSpray ESI interface supported with nanoBooster dopant addition technology (Bruker Daltonik GmbH). Dried glycopeptides were redissolved in 20 μl of ultrapure water and were diluted three times before loading onto Acclaim PepMap C18 trap column (5 × 0.3 mm, 5 μm, 100 Å, Thermo Fisher Scientific). On the trap column, analytes were desalted for 3 min with 0.1% formic acid (FA) (v/v) at a 40 μl/min flow rate and then transferred to the analytical column. Before each sample was injected, the trap column was washed with 20 μl of isopropanol/ACN (25:75, v/v) followed by the same volume of 95% ACN (v/v). Separation of the glycopeptides was performed based on their peptide backbone and was done on a HALO C18 column (150 × 0.1 mm, 2.7 μm, Advanced Materials Technology). Column temperature was set to 30 °C, and flow rate was 1 μl/min. Gradient started from 0% eluent B (0.1% FA in 80% ACN, v/v) and 100% eluent A (0.1% FA, v/v) and was increased from 0% to 25% B during the first 3.5 min and kept at 25% for another 1.5 min. During the next 10 min, eluent B was further increased to 36%, ending with 100% eluent B 6 min wash, and a total 21 min gradient. After separation, the column was equilibrated with 100% eluent A for 6 min.

The mass spectrometer was operated in a positive ion mode with rolling average of spectra acquisition rate at 2 × 0.5 Hz and in m/z range 200 to 2000. Capillary voltage was set to 3.3 kV, and drying gas temperature was 200 °C at 3.5 l/min. Nitrogen nebulizing gas enhanced with ACN was provided at 0.3 bar. Nano-LC-MS system was operated under otofControl, MassLynx, and Compass HyStar software (all v.4.1).

For the annotation of the C3 glycopeptides and their MS/MS structural characterization, the same settings were used with some modifications. Fragmentation spectra of the glycopeptides were recorded in a stepwise manner, with collision energies ranging from 17 to 40 eV linearly dependent on the charge state and m/z value of the selected precursor. These values were applied for 20% of the MS2 events and doubled for the remaining time in order to acquire both, glycan specific and peptide specific fragments (28). Top three precursors were automatically selected, and isolation windows were set at otofControl default values depending on the precursor m/z value. Additional fragmentation of the glycopeptides in order to fully characterize their composition was performed on Orbitrap Exploris 240 mass spectrometer (Thermo Fisher Scientific), with full scan properties as follows: resolution 60,000, m/z range 600 to 2000, data-dependent MS scan resolution 45,000, and HCD collision energy set at 15%.

C3 glycopeptides were manually annotated using MS and MS/MS spectra generated in the data-dependent acquisition mode. A list of expected C3 glycoforms for each glycosylation site was prepared based on the literature data (16, 17, 18). Assumed glycopeptides were assigned based on their theoretical m/z value calculated for multiply charged protonated ions and then confirmed using the MS/MS spectra. For manual interpretation of the fragmentation spectra, Compass DataAnalysis (v.4.1) and GlycoWorkbench 2.0 software were employed (29), the latter being operated under permissive free software licenses. During the annotation process, all literature data on C3 glycan structures as well as all potential perils of MS-based composition annotation were taken into account. Summed mass spectra of the peak clusters were manually searched for potentially additional glycoforms, and the whole MS/MS spectra were searched for glycopeptide diagnostic ions such as GlcNAc_1_Man_1_ with the same goal. Results of the manual annotation are presented in supplemental Table S2.

### Proteomic Data Analysis

To evaluate the efficacy of our C3 enrichment protocol and to identify potentially coenriched glycoproteins in the Con A eluate, we performed a proteomic analysis using MaxQuant software (version 1.6.10.43). Human reference proteome (release version: 2019_07, ProteomeID: UP000005640) containing 75,069 protein sequence entries was downloaded from Uniprot and uploaded to MaxQuant. Instrument type was set to Bruker Q-TOF, and all the peak picking parameters (Maxquant group specific parameters) were left at default settings, except for the first search peptide tolerance which was set to 0.1 Da. Main peptide tolerance search remained 0.006 Da, and fragment mass tolerance was set at default value of 50 ppm. Enzyme was set to GluC with specific cleavages and with maximum of two misses. At both protein and peptide spectrum match level false discovery rate was set to 1% and was determined by the target-decoy approach. Oxidation (M), acetyl (Protein N-term), and carbamidomethyl (C) were set as variable modifications. Using the MS^1^ level data, identified proteins were quantified summing up the extracted ion currents of all the isotopic clusters associated with identified peptide sequence. Relative abundance of each identified protein was expressed as percentage of total intensity (protein groups table of the MaxQuant output).

### Glycoproteomic Data Extraction and Processing

After the annotation, two chromatographic peak clusters with three glycoforms each were defined. Data extraction was performed using LaCy Tools as described previously (30). LaCy Tools data processing package (v.1.0.1, b.9), operating under free software license, was used for fast automatic peak integration (31). Prior to the quantification of the glycoproteomic data, original data files were first converted to mzXML open data format by ProteoWizard MSConvert tool (version 3.0.19208) (32). Chromatograms were aligned based on the retention times of five most abundant glycopeptide signals. Targeted peak integration was performed on triply, quadruply, and quintuply charged species. Summed spectra were integrated to include at least 90% of the theoretical isotopic pattern. Extracted data quality control parameters (mass accuracy, deviation from the theoretical isotopic pattern, and signal to noise ratio) were automatically calculated for each analyte of every sample. Quality of the data was assessed using average quality control values calculated from all analyzed samples, for every glycoform and charge state separately. Inclusion criteria were set as follows: mass accuracy below 100 ppm, isotopic pattern quality below 25%, and signal to noise ratio above 8. For all the glycoforms, only the charge states compliant with these three criteria were summed and quantified.

### Glycoproteomic Statistical Data Analysis

Measurements were logit-transformed and then batch corrected using ComBat method (R package sva) (33). Plate number of the analyzed samples, which represented the laboratory source of variation, was modeled as a batch covariate. Estimated batch effects were then subtracted from the logit-transformed measurements reducing the introduced experimental noise. Assessment of T1D association with C3 N-glycome was done using linear mixed modeling with glycoform area as dependent variable and the presence of T1D as independent variable. To remove the effect of age and sex differences between the case and control groups, these variables were included as additional covariates. Moreover, to account for genetic similarity between siblings, siblings from the same family were grouped and their family ID included as random intercept. The results are presented in odds ratio (T1D *versus* control) for each glycoform measured. Before any statistical modeling, glycoform variables were normalized to total chromatogram area for each glycosylation site. Considering multiple tests performed, false discovery rate was controlled by the Benjamini–Hochberg method with *p*-value <0.05 considered as significant (34).

C3 N-glycan profile was then assessed for its ability to differentiate individuals with T1D from their healthy siblings. Interrelationship of T1D status and C3 N-glycome was estimated on all siblings using logistic mixed model elastic net regression (α = 1, λ = 0.02), by comparing AUC of two receiver operating characteristic (ROC) curves (R packages glmnet (35) and pROC (36)) obtained from two predictive models. First (null) model used only sex and age as predictors of disease status, while second (full) model used these variables and all the glycoforms. To avoid overfitting, 5-fold cross validation was used. Estimated AUCs of ROC curves were compared using bootstrapping (2000 replicates).

The data were analyzed and visualized using the R programming language (version 3.5.2) and Microsoft Excel 2016 (Microsoft Corp).

## Results

### Development of High-Throughput Human C3 N-Glycosylation Analysis

**Fig. 1:**
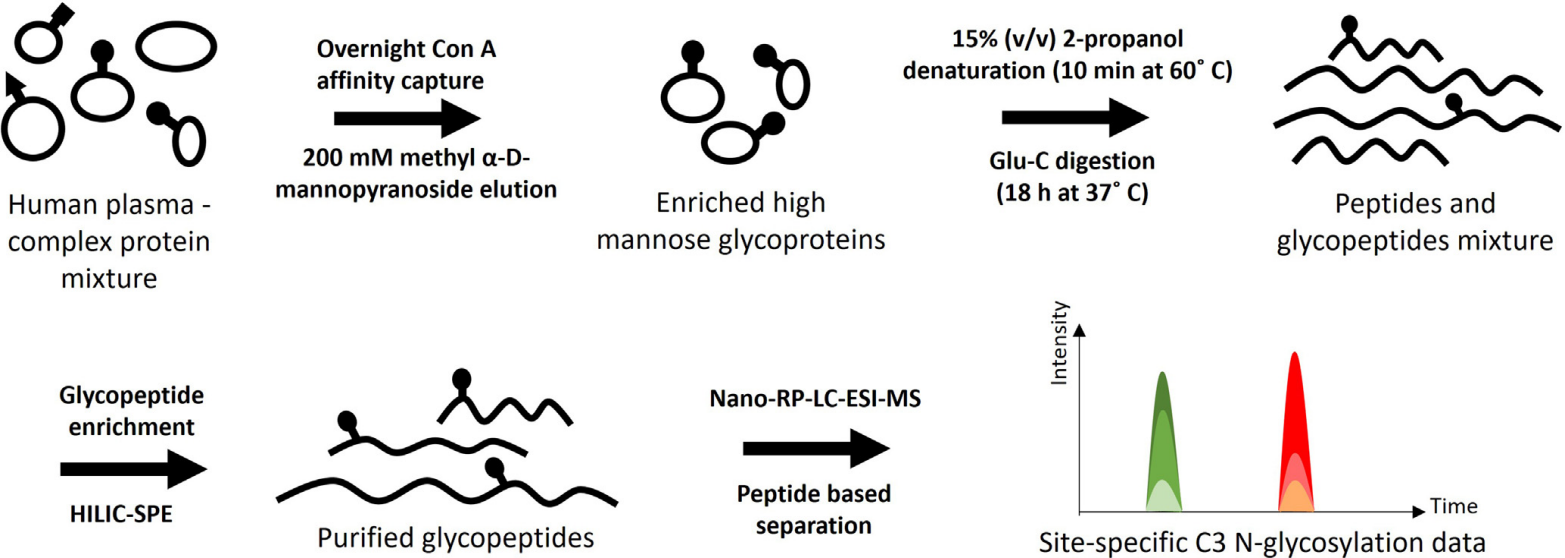
**Schematic glycoproteomic workflow for a high-throughput and site-specific N-glycosylation LC-MS analysis of human C3.** C3, complement component 3; HILIC-SPE, hydrophilic interaction chromatography–based solid-phase extraction; RP-LC-ESI-MS, reverse-phase liquid chromatography electrospray ionization mass spectrometry.

Reduction of sample complexity and enrichment of glycoproteins is an essential first step of glycoproteomic analysis, and one of the most commonly used enrichment approaches is lectin affinity chromatography (37). Since C3 contains exclusively high-mannose glycans, for the lectin mediator we chose Con A, a commonly used lectin which preferentially recognizes glycans presenting α-mannose-containing cores, and to lesser extent glucosyl oligosaccharides (38, 39). This approach enriches not only intact C3 but also its smaller fragments which are also glycosylated, such as C3b. Con A sepharose affinity media enables fast high-throughput and cost-effective C3 enrichment from human plasma in a 96-well format. We found that digestion of eluted glycoproteins with endoproteinase Glu-C, instead of commonly used trypsin, generated adequate peptide sequence lengths for C3. According to our proteomic data analysis, C3 accounted for on average of 11% of all the proteins in the Con A eluate (supplemental Table S3), which was adequate for the following analysis of its site-specific N-glycosylation. Figure 1 shows the complete schematic workflow for the high-throughput human C3 N-glycosylation analysis.

To determine the technical variation of the developed glycoproteomic workflow, we measured a CV of the pooled plasma standard technical replicates. Repeatability was calculated using octuplicates across a 96-well plate, and within-laboratory interday reproducibility was assessed using three octaplicates from three separately performed sample preparations week after week. Median CV was below 12% for all the glycoforms analyzed (supplemental Table S4). Increased variation was observed for the monoglucosylated glycoform, while the median CV for all the other glycoforms was below 5%.

### C3 N-Glycosylation Profiling

N-glycosylation profiling of human C3 was first done on a plasma standard made from pooled plasma of the population for the intraindividual temporal stability study. A list of expected C3 glycoforms for each glycosylation site was prepared based on previously reported glycan structures (18). Human C3 has three potential N-glycosylation sites, but only two are occupied and contain different high-mannose glycans (26, 40). Glycoforms were identified by m/z values calculated from the mass of each Glu-C digested peptide carrying N-glycosylation site and the mass of its corresponding glycans and then confirmed using the fragmentation spectra. All genetic variants of human C3 were taken into consideration, and none affected the sequence of Glu-C digested N-linked glycopeptides. In total, six exclusively high-mannose glycoforms were identified, three per site. This confirmed the previous reports, as we have not found any other glycoform present. The glycan composition of each site and the abbreviations used for naming the C3 glycoforms are presented in Table 1. Based on the position of the glycosylation sites, the first site is named C3.Asn85 and the second C3.Asn939. Glycoform abbreviation is composed of the site name and number of hexoses (H) and N-acetylglucosamines (N) it contains.

**Table 1:** Glu-C digested peptide sequence of each site, its glycan composition, and glycoform abbreviation

N-glycosylation site	Peptide sequence	Glycan composition	Glycoform abbreviation
Asn 85	KTVLTPATNHMGN∗VTFTIPANRE	Man_5_GlcNAc_2_	C3.Asn85-N2H5
		Man_6_GlcNAc_2_	C3.Asn85-N2H6
		Man_7_GlcNAc_2_	C3.Asn85-N2H7
Asn 939	GIRMN∗KTVAVRTLDPE	Man_8_GlcNAc_2_	C3.Asn939-N2H8
		Man_9_GlcNAc_2_	C3.Asn939-N2H9
		Glc_1_Man_9_GlcNAc_2_	C3.Asn939-N2H10

N-linked asparagine is marked with ∗.

Abbreviations: C3.Asn85, first site; C3.Asn939, second site; H, hexose; N – N-Acetylglucosamine.

**Fig. 2:**
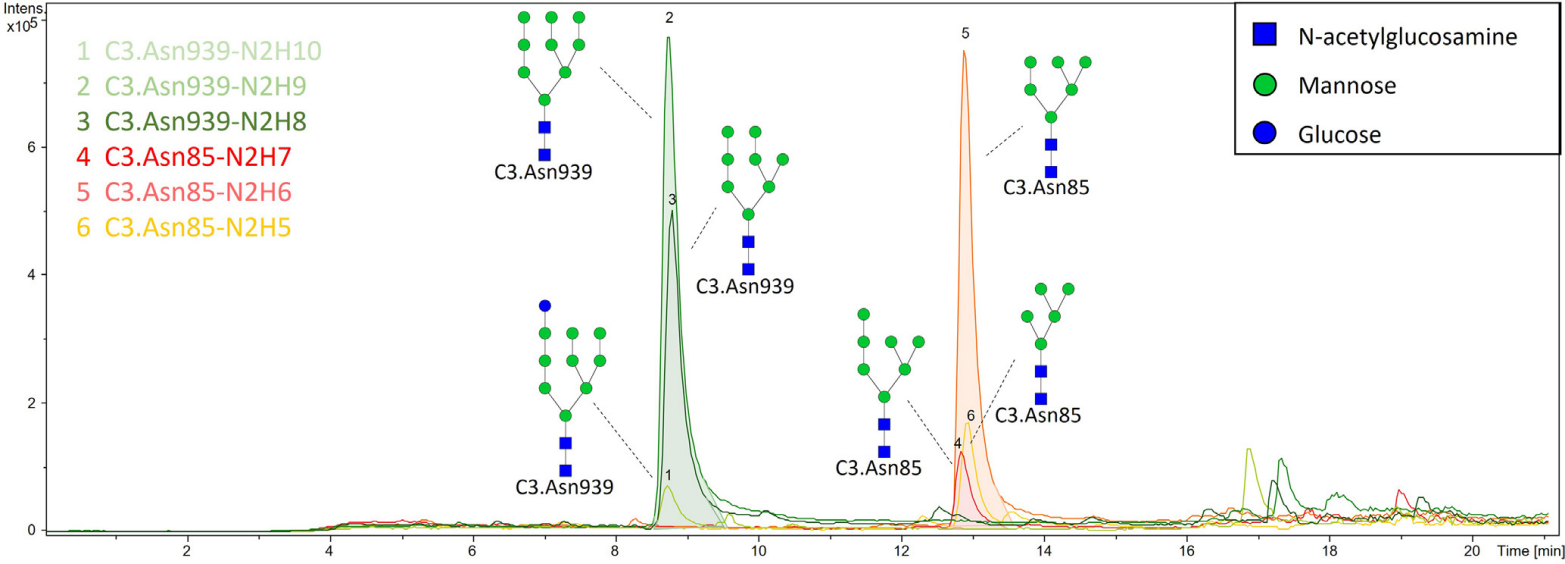
**Result of LC-MS C3 glycosylation analysis by newly developed method: representative chromatogram with extracted ion traces of all six C3 glycoforms from its two N-glycosylation sites**.

**Fig. 3:**
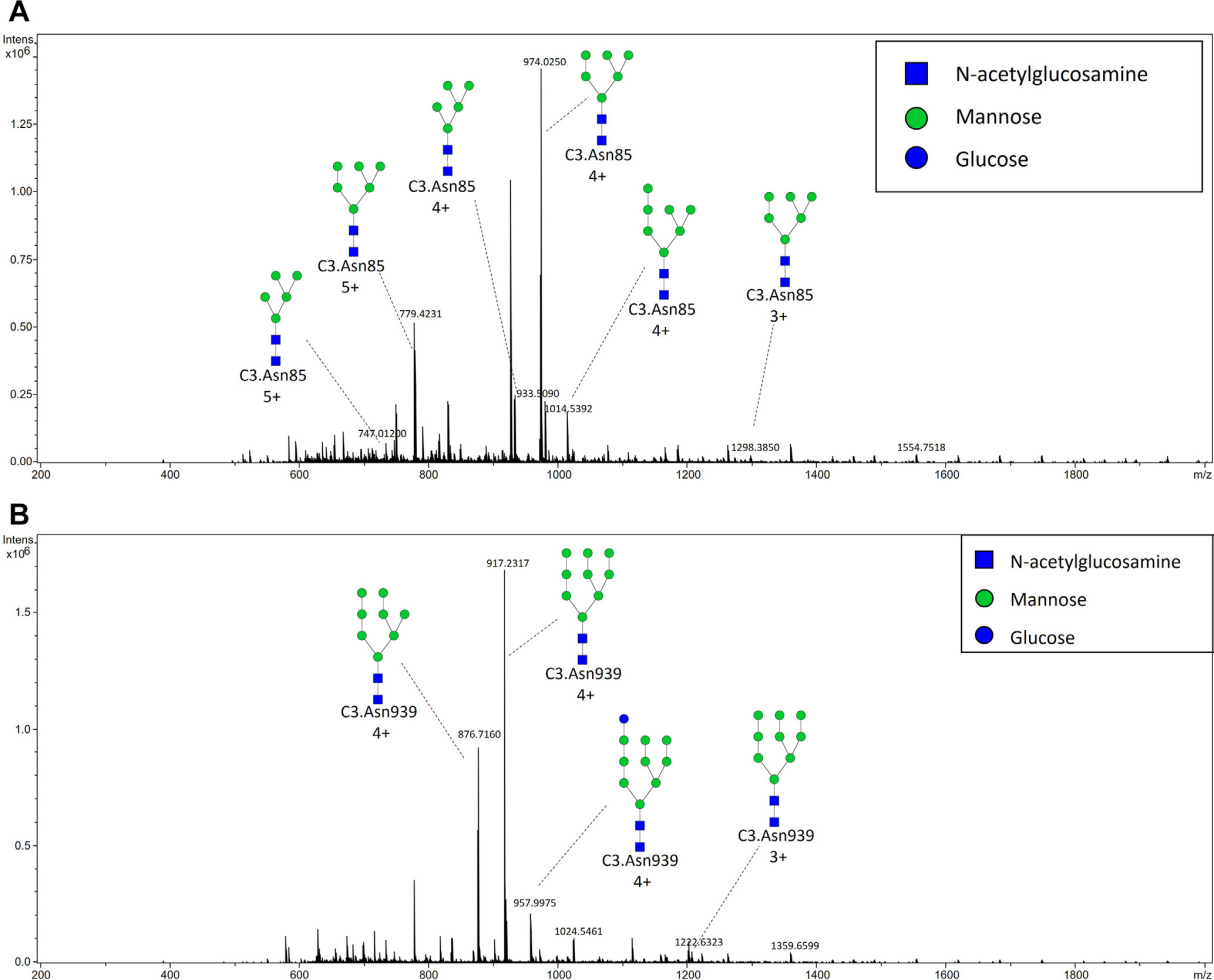
**Typical summed mass spectrum.***A*, C3.Asn85 peak cluster with annotated triply [M+3H]^3+^, quadruply [M+4H]^4+^, and quintuply [M+5H]^5+^ charged glycopeptide ions. *B*, C3.Asn939 peak cluster with annotated triply [M+3H]^3+^ and quadruply [M+4H]^4+^ charged glycopeptide ions.

A representative chromatogram of human C3 glycopeptides with extracted ion traces forming two clusters is shown in Figure 2, and a typical summed mass spectra for one of the peak clusters is depicted in Figure 3, *A* and *B*. Supplemental Fig. S1 shows the base peak intensity chromatogram together with extracted ion traces of C3 glycoforms and indicates a good chromatographic separation, with C3 glycopeptides being highly abundant in their peaks. Although the Con A eluate is still a very complex mixture of different glycoproteins, our fast 21-min reversed-phase nano-LC analysis efficiently separates Glu-C peptides, with minimum co-elution from non-C3 peptides complicating the mass spectra. That was not the case when the digestion was performed with trypsin, because one of the N-glycosylation site’s peptide tryptic sequence consisted of only three amino acids (supplemental Table S5), disabling the coherent interaction with C18 column used for separation and causing analysis problems.

**Fig. 4:**
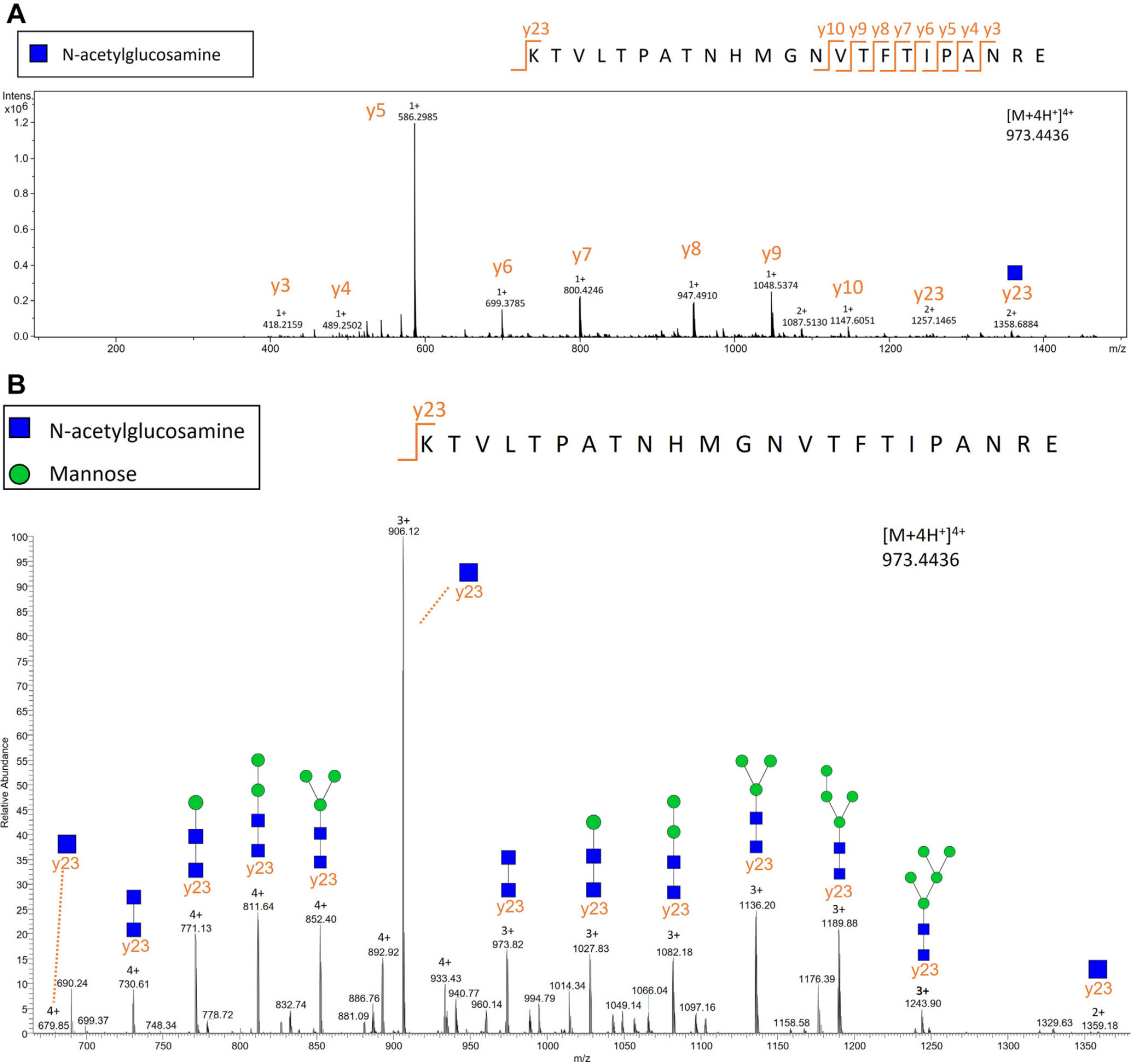
**MS/MS fragmentation spectrum of C3.Asn85-N2H6.***A*, fragmentation pattern of the peptide part of C3.Asn85-N2H6. *B*, fragmentation pattern of the glycan part of C3.Asn85-N2H6. C3, complement component 3; H, hexose; N, N-Acetylglucosamine.

All of the six glycoforms identified were confirmed in the MS/MS analysis that followed. Fragmentation spectra were acquired by tuning energy stepping collision induced dissociation of glycopeptides, enabling the acquisition of glycan and peptide-specific fragments within a single tandem MS spectrum. Interestingly, C3.Asn85 glycoforms predominantly dissociated on the peptide level, while C3.Asn939 glycopeptides were characterized from mostly glycan part fragments. Collision-induced dissociation of C3.Asn85 glycopeptides primarily yielded y-type ions, whereas for the C3.Asn939 glycoforms, combinations of y-type and b-type ions were generated. To better characterize the glycosidic fragmentation, we additionally performed HCD collision-induced dissociation study with satisfying results. Figure 4 shows typical example fragmentation spectra for C3.Asn85-N2H6 on both peptide and glycan level. The rest of the glycoforms showed similar fragmentation patterns, and their MS/MS spectra are presented in supplemental Fig. S2.

### Intraindividual Temporal Stability Study

Although the structural composition of C3 N-glycome was already known, its stability over time within an individual, as well as interindividual differences, had not been studied. To better understand C3 N-glycan profile temporal consistency, samples from 14 healthy male young adults were analyzed at three time points: at the beginning of the study and after 6 and 10 weeks. Considering the fact that C3 half-life in plasma is relatively short (approximately 72 h) and that its cleavage products are even shorter lived (41, 42, 43), these time gaps should secure the analysis of newly synthesized protein at each sample collection point.

Temporal stability was assessed by calculating intraindividual and interindividual CV for all the glycoforms. Intraindividual CV was measured from longitudinal samples of each subject, while the interindividual CV was computed from all samples within each time point. Within-laboratory interday reproducibility assessed previously (supplemental Table S4) served as the CV of the method itself. The data presented in Table 2 show that median CV between participants is above both intraindividual and method variation, demonstrating that the interindividual differences are more pronounced than changes within the individual, for all the glycoforms. Even though glycoforms C3.Asn85-N2H5 and C3.Asn85-N2H6 had somewhat comparable intraindividual and interindividual variation, the rest of the glycoforms clearly manifested larger variation among individuals (supplemental Fig. S3). The small method reproducibility CV guarantees that these distinctions did not come from the method itself, indicating the perseverance of the C3 N-glycome under physiological conditions.

**Table 2:** Temporal stability of C3 N-glycosylation in healthy individuals over 10 weeks

Glycoform	Intraindividual CV (%)	Interindividual CV (%)	Method reproducibility CV (%)
C3.Asn85-N2H5	10.43	11.14	3.74
C3.Asn85-N2H6	4.15	4.21	0.78
C3.Asn85-N2H7	6.89	16.20	3.28
C3.Asn939-N2H8	4.61	9.34	1.78
C3.Asn939-N2H9	2.16	4.52	1.23
C3.Asn939-N2H10	17.08	20.38	11.27

Intraindividual coefficient of variation (CV) calculated from longitudinal samples of each participant, inter-individual CV from all participants’ samples within each time point, and method interday reproducibility CV. Median CVs are shown.

Abbreviations: C3Asn85, first site; C3.Asn939, second site; H, hexose; N, N-Acetylglucosamine.

### C3 N-Glycome in Type 1 Diabetes

**Fig. 5:**
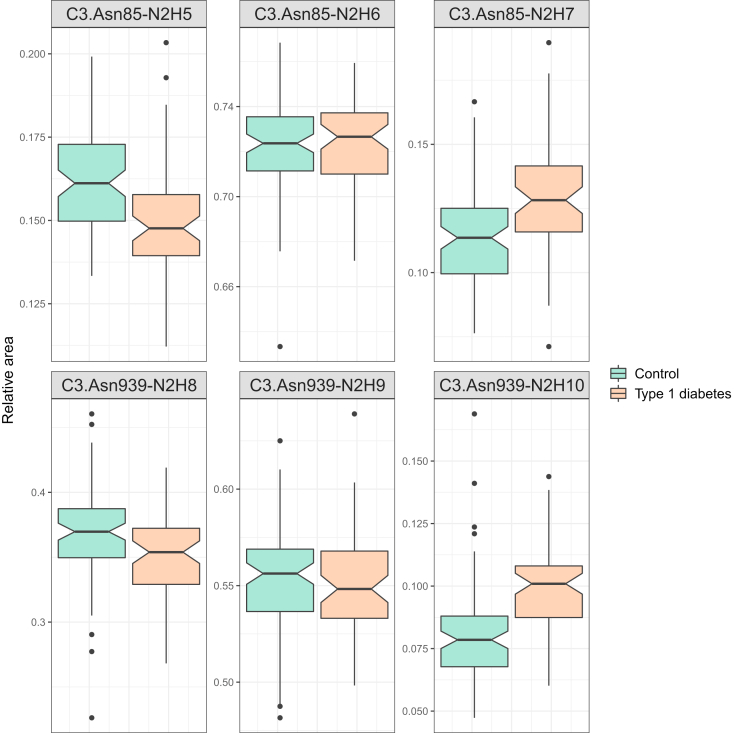
**Box plots of batch corrected data showing significant increases in the proportion of unprocessed glycan structures on both C3 N-glycosylation sites for type 1 diabetes compared to sibling controls.***Dots* are outliers. C3, complement component 3; C3.Asn85, site on Asn 85; C3.Asn939, site on Asn 939; H, hexose; N, N-Acetylglucosamine.

As C3 N-glycosylation proved to be stable within a healthy individual, its potential use as a biomarker was enabled. To investigate the C3 N-glycan profile as a diagnostic tool, we applied our method to samples from children and adolescents diagnosed with T1D, together with those from their healthy siblings. The results are presented in supplemental Table S6. Linear regression showed that the relative proportions of four glycoforms were significantly changed in T1D cases compared to the control group (Table 3). Interestingly, both of the sites exhibited a shift toward more unprocessed glycan structures, *i.e.*, structures with more mannose units (Fig. 5). On the C3.Asn85 site, relative abundance of Man_7_GlcNAc_2_ was significantly higher (OR = 1.15, *p* = 5.43 × 10^−5^) at the expense of lower levels of Man_5_GlcNAc_2_ (OR = 0.89, *p* = 7.82 × 10^−8^), while the level of Man_6_GlcNAc_2_ remained unchanged. On the C3.Asn939 site, a significantly larger proportion of Glc_1_Man_9_GlcNAc_2_ (OR = 1.28, *p* = 1.78 × 10^−8^) was observed at the cost of Man_8_GlcNAc_2_ levels (OR = 0.91, *p* = 1.44 × 10^−3^), while the level of Man_9_GlcNAc_2_ remained unchanged. An increase in the proportion of Man_7_GlcNAc_2_ was also observed in our previous study of the total plasma N-glycome.

**Table 3:** Calculated effects of type 1 diabetes on C3 glycoforms and their respective unadjusted and adjusted *p*-values

Glycoform	Odds ratio (95% CI)	Unadjusted *p*-value	BH-adjusted *p*-value
**C3.Asn85-N2H5**	**0.89 (0.85–0.92)**	**2.61 × 10**^**−8**^	**7.82 × 10**^**−8**^
C3.Asn85-N2H6	1.00 (0.97–1.04)	7.94 × 10^−1^	7.94 × 10^−1^
**C3.Asn85-N2H7**	**1.15 (1.08–1.22)**	**2.71 × 10**^**−5**^	**5.43 × 10**^**−5**^
**C3.Asn939-N2H8**	**0.91 (0.87–0.96)**	**9.62 × 10**^**−4**^	**1.44 × 10**^**−3**^
C3.Asn939-N2H9	1.01 (0.97–1.05)	6.17 × 10^−1^	7.41 × 10^−1^
**C3.Asn939-N2H10**	**1.28 (1.19–1.38)**	**2.97 × 10**^**−9**^	**1.78 × 10**^**−8**^

False discovery rate (FDR) was controlled by the Benjamini–Hochberg (BH) method with *p*-value <0.05 considered as significant. Statistically different glycoforms are marked in bold.

Abbreviations: C3.Asn85, first site; C3.Asn939, second site; CI, confidence interval; H, hexose; N, N-Acetylglucosamine.

**Fig. 6:**
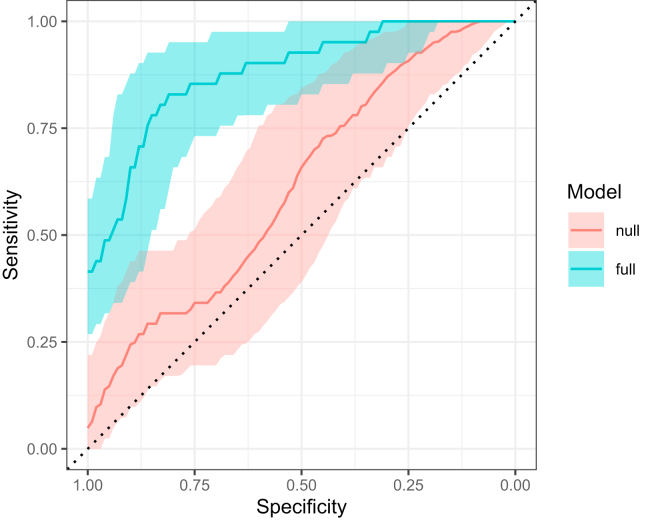
**Receiver operating characteristic (ROC) curve analysis of two models for prediction of type 1 diabetes.** Null model (AUC 0.614) uses only sex and age as predictors, while full model (AUC 0.879) includes C3 glycoforms. *Shaded area* around lines presents 95% confidence interval. AUC, area under receiver-operator curve.

To evaluate the ability of the C3 N-glycan profile to differentiate individuals with T1D from their healthy siblings, a glycan-based discriminative model was built using logistic mixed model elastic net regression. In the discriminative model, all of the C3 glycoforms were used as predictors, and the models were assessed using a ROC curve analysis (Fig. 6). A model based only on age and sex (null) did not have significant discriminative power (AUC 0.614, 95% CI 0.497–0.719). Addition of C3 glycoforms into the model (full) increased the discriminative power considerably (AUC 0.879, 95% CI 0.801–0.936), indicating their predictive power in T1D risk assessment.

## Discussion

In this study, we aimed to characterize the complement component C3 N-glycosylation profile in children and adolescents at the onset of T1D. For this purpose, it was first necessary to develop a novel high-throughput method for C3 plasma enrichment and MS analysis of its N-glycosylation. Using our method, we showed that C3 N-glycome significantly differed between children newly diagnosed with T1D and their healthy siblings, potentially allowing it to be used for T1D risk assessment. This study confirmed our previous findings of total plasma N-glycosylation changes of high-mannose glycan levels in a cohort of recent onset T1D cases. Moreover, as the proportion of glycans from total plasma N-glycome could have been impacted by protein concentration, our study empowered the conclusion that observed changes were not solely the consequence of this phenomenon but were also evident in a glycome of a single protein. As the research population consisted of young individuals at the beginning of the disease, the observed glycosylation changes may be associated with the disease onset. Additionally, a young population is less influenced by other comorbidities seen in the adult population, therefore enabling more precise research of specific changes accompanying the disease. Although it could be argued that studying siblings lessens the differences between cases and controls, incorporating both genetic and environmental similarity allows for any differences observed to be more likely attributable to disease processes. It should also be stated that this kind of study design limits us in our conclusions whether the relative distributions of C3 glycoforms are indeed a risk factor for T1D *per se*, or these changes are merely secondary to disease onset.

To the best of our knowledge, this is the first study of C3 N-glycosylation changes in any disease, and the first MS-based structural identification of C3 glycoforms and verification of its glycosylation sites. Our study confirmed the previous reports of C3 having only two glycosylation sites occupied (out of a potential three), linking only high-mannose glycans (18, 26, 40). This is also the first study of C3 N-glycosylation intraindividual temporal stability in healthy individuals, which is a requirement for evaluation of its diagnostic potential.

Although many protein biomarkers currently in clinical use are glycoproteins, their N-glycosylation profile is usually poorly examined, at best. Given the fact that the glycome is considerably influenced by the environment, reflecting individual lifestyles that go beyond the inherited genetic background, changes in N-glycosylation could provide us with valuable information about ongoing pathological conditions (44). Here, we demonstrated the temporal stability of the C3 N-glycome, allowing its consideration as a biomarker. C3 glycosylation proved to be mostly stable under physiological conditions, with its intraindividual variation being smaller than the variation between individuals. In order to remove the effect of female hormonal cycle and age, subjects were all young males, so our results represent the minimum interindividual variation.

High-mannose structures are often defined as immature N-glycans, as they skip the late processing steps in the Golgi that the majority of N-glycans receive (9). Aberrant high-mannose N-glycan abundancies in humans have been linked to tumor progression, especially in breast and prostate cancer (45, 46). Previous studies of plasma N-glycosylation found significant associations of high-mannose N-glycans and renal function decline in T1D subjects (12, 13), and an increase in MBL has also been reported (5). Taking into consideration that C3 has only a high-mannose N-glycome and that there is an increasing evidence that the complement system plays an important role in T1D (4), we hypothesized that C3 N-glycosylation aberrations could provide a valuable biomarker of T1D pathophysiology. Our study confirms this hypothesis, as we found four C3 N-glycoforms that were significantly changed in T1D cases compared to the control group. For both sites, an increase in the proportion of more unprocessed glycans was observed in children with T1D relative to their healthy siblings: more Man_7_GlcNAc_2_ increase and less Man_5_GlcNAc_2_ were found on the C3.Asn85 site, while the C3.Asn939 site was characterized by significantly higher Glc_1_Man_9_GlcNAc_2_ and lower Man_8_GlcNAc_2_ levels. Proportions of Man_6_GlcNAc_2_ and Man_9_GlcNAc_2_ remained unchanged. Interestingly, the biggest change observed was in monoglucosylated Glc_1_Man_9_GlcNAc_2_ (OR = 1.28, *p* = 1.78 × 10^−8^). Findings of these significant changes in C3 N-glycome allowed us to develop a glycan-based predictive model for T1D. With its solid discriminative power (AUC 0.879), our model could help in identifying individuals at higher risk of developing the disease. This model fairly compares to already reported plasma N-glycome model (8) but lacks the disadvantages of different protein concentration impact.

Whether the observed N-glycosylation changes are causative or reflective of a disease status remains an open question. The increase of unprocessed glycans in T1D could possibly be explained by the fact that besides the main production in liver, kidneys (47), and pancreatic islets (48) also highly express C3, contributing to its total production with about 4% each. Specifically, this local C3 aberrant glycan profile could be explained by (or accounted for) ER stress typical for renal and pancreatic cells in T1D (49, 50). Namely, pancreatic beta cells are highly sensitive to ER stress because of their large insulin secretion. In T1D, functioning beta cells experience intensifying and unresolvable ER stress as they compensate for neighboring cells that have become dysfunctional, further threatening their own survival (49, 51). On the other hand, high glucose–induced ER stress in renal tubular epithelial cells is considered as one of the key drivers of disease progression in diabetic nephropathy (50). N-glycans have already been proposed as potential descriptors of the ER stress, and a shift toward more unprocessed of the high-mannose glycans was found significant *in vitro* (52). Therefore, observed glycosylation changes are likely associated with the presence of ER stress and could possibly be a marker of its progression and the progression of T1D itself. Moreover, given the fact that autoimmune destruction of insulin producing beta cells can begin years before clinical diagnosis (53), changes in C3 N-glycome are possibly present even prior to onset of symptoms. The C3 N-glycome profile analysis could thus be used as a diagnostic tool for monitoring this disease. Our glycan-based predictive model showed solid discriminative power and merging with clinical data could further optimize discrimination, suggesting that the C3 N-glycome profile could be a novel risk factor in T1D.

## Data Availability

MaxQuant data and all raw data files are publicly available on the PRIDE archive (http://www.ebi.ac.uk/pride) under the identifier: PXD034083 and PXD035126. The mass spectrometry proteomics data have been deposited to the ProteomeXchange Consortium *via* the PRIDE partner repository with the dataset identifiers PXD034083 and PXD035126.

## Supplemental data

This article contains supplemental data.

## Conflict of interest

G. L. is the founder and owner, and M. N. is an employee of Genos Glycoscience Research Laboratory, a company that specializes in high-throughput glycomics and has several patents in this field. The remaining authors declare that the research was conducted in the absence of any commercial or financial relationships that could be construed as a potential conflict of interest.
