# Supplementary material for: High-Throughput Human Complement C3 N-Glycoprofiling Identifies Markers of Early Onset Type 1 Diabetes Mellitus in Children
Source: Mol Cell Proteomics. 2022 Aug 27;21(10):100407. doi: 10.1016/j.mcpro.2022.100407 (PMC9538898; doi:10.1016/j.mcpro.2022.100407)
Supplement: Supplemental Tables S1, S4, S5 and Figures S1–S3 [file mmc4.docx]

**Supplemental Data**

**High-throughput human complement C3 N-glycoprofiling as a marker of early onset type 1 diabetes mellitus in children**

**Dinko Šoić^1^, Toma Keser^1^, Jerko Štambuk^2^, Domagoj Kifer^1^, Flemming Pociot^3,4^, Gordan Lauc^1,2^, Grant Morahan^5^, Mislav Novokmet^2^, and Olga Gornik^1*^**

This file includes:

Supplemental Tables 1 to 6

Supplemental Figures 1 to 3

**Supplemental tables**

**Table S1** Descriptive data of the pilot study (children and adolescents newly diagnosed with T1D and their unaffected siblings).

|  | T1D | Control |
| --- | --- | --- |
| Number of patients (N) | 61 | 84 |
| Age (years) | 10 (1-16) | 11 (4-22) |
| Female sex (N, %) | 29 (47.5 %) | 39 (46.4 %) |
| T1D - type 1 diabetes | | |

**Table S2** Manually annotated spectra based on MS/MS data for all six C3 glycopeptides. The annotation was done according to following criteria: fragmentational spectra, same retention time window, m/z value, presence of more than one charge state. Available as a separate file.

**Table S3.** MaxQuant analysis of the enriched C3 eluate following HILIC-SPE purification protocol. Reference proteome UPID: UP000005640. Available as a separate file.

**Table S4.** Repeatability of developed workflow. Calculated median CV values for octaplicates from three separately performed sample preparations. C3.Asn85 – site Asn 85, C3.Asn939 – site Asn 939, N – N-Acetylglucosamine, H – hexose.

|  | Glycoform | C3.Asn85-N2H5 | C3.Asn85-N2H6 | C3.Asn85-N2H7 | C3.Asn939-N2H8 | C3.Asn939-N2H9 | C3.Asn939-N2H10 |
| --- | --- | --- | --- | --- | --- | --- | --- |
| Sample preparation 1 | Average relative area | 0.136 | 0.749 | 0.116 | 0.341 | 0.573 | 0.086 |
|  | SD | 0.013 | 0.018 | 0.009 | 0.017 | 0.020 | 0.022 |
|  | **CV (%)** | **9.833** | **2.396** | **7.393** | **5.104** | **3.455** | **25.199** |
|  | | | | | | | |
| Sample preparation 2 | Average relative area | 0.130 | 0.754 | 0.116 | 0.328 | 0.599 | 0.073 |
|  | SD | 0.005 | 0.006 | 0.004 | 0.006 | 0.005 | 0.002 |
|  | **CV (%)** | **3.742** | **0.775** | **3.279** | **1.781** | **0.821** | **2.811** |
|  | | | | | | | |
| Sample preparation 3 | Average relative area | 0.138 | 0.735 | 0.128 | 0.323 | 0.602 | 0.075 |
|  | SD | 0.003 | 0.002 | 0.002 | 0.006 | 0.007 | 0.008 |
|  | **CV (%)** | **1.964** | **0.301** | **1.656** | **1.708** | **1.231** | **11.277** |
| **median CV (%)** |  | **3.742** | **0.775** | **3.279** | **1.781** | **1.231** | **11.277** |

**Table S5.** Peptide sequence for C3 N-glycosylation sites after digestion with Glu-C and trypsin. N-linked asparagine is marked with *. C3.Asn85 –site Asn 85, C3.Asn939 –site Asn 939.

| N-glycosylation site | Enzyme | Peptide sequence |
| --- | --- | --- |
| C3.Asn85 | Glu-C | KTVLTPATNHMGN*VTFTIPANRE |
|  | Trypsin | TVLTPATNHMGN*VTFTIPANR |
| C3.Asn939 | Glu-C | GIRMN*KTVAVRTLDPE |
|  | Trypsin | MN*K |

**Table S6.** Clinical data and relative area of C3 glycoforms for every sample used in the pilot study. Available as a separate file.

**Supplemental figures**

**
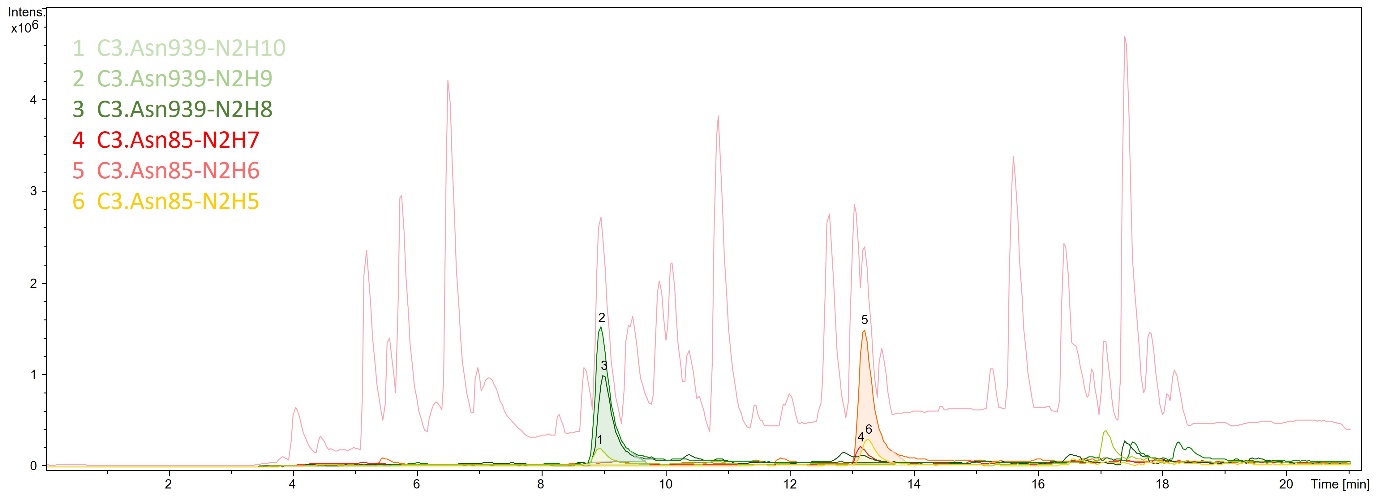
**

**Figure S1** An overlapped chromatogram including BPI and extracted ion traces of all six C3 glycoforms.

**A**

**
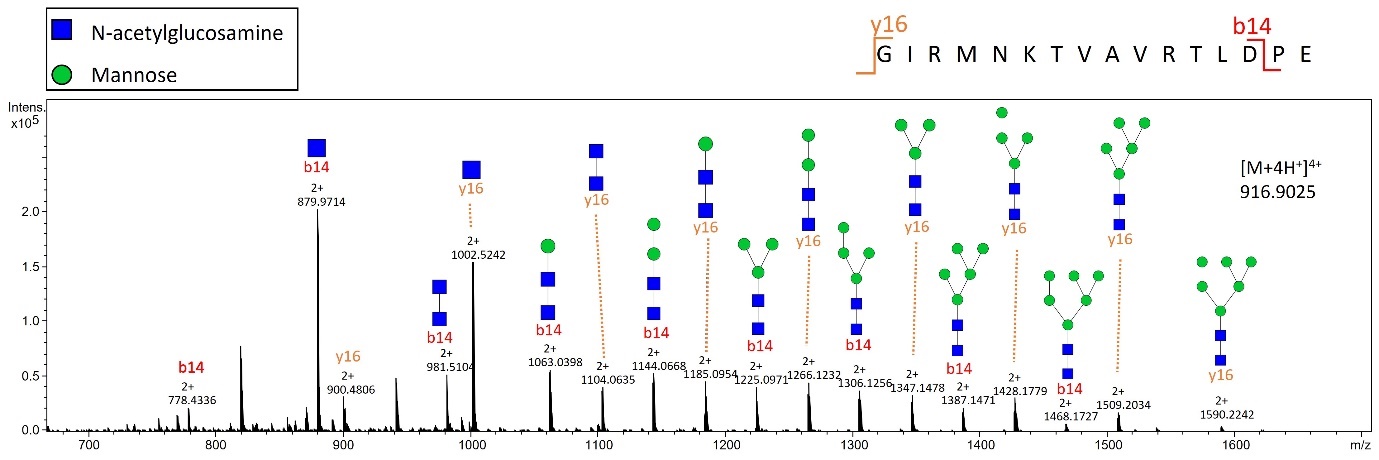
**

**B
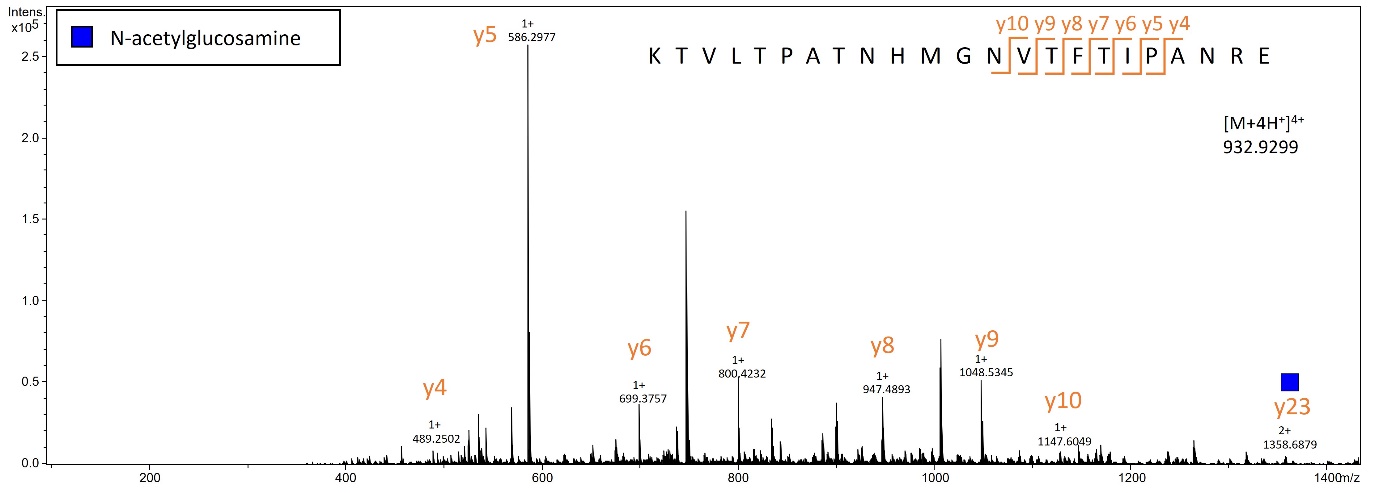
**

**C
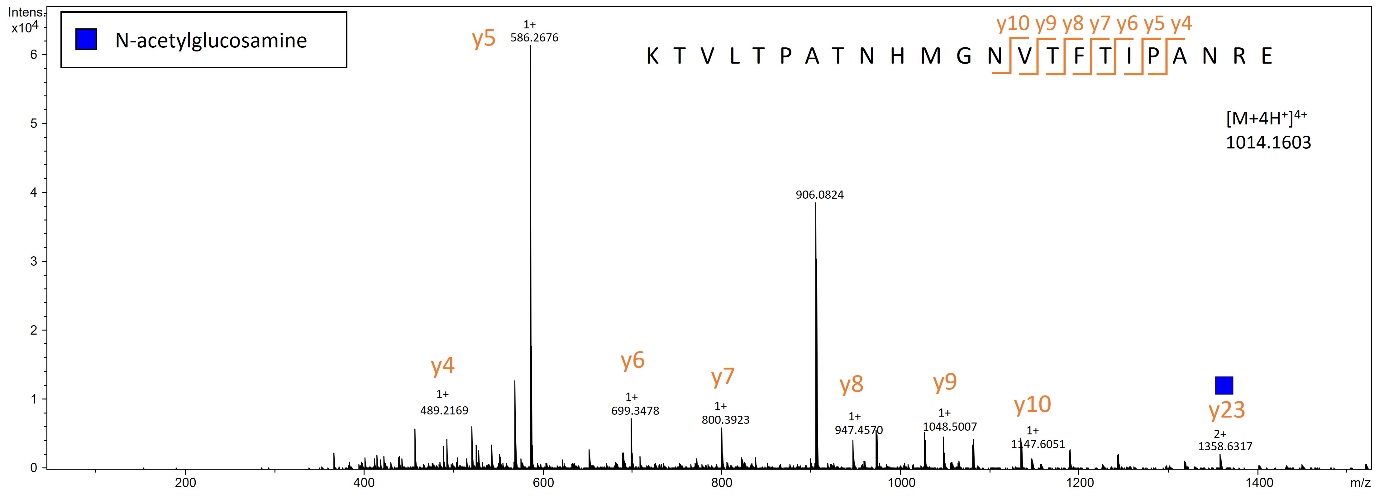
**

**D
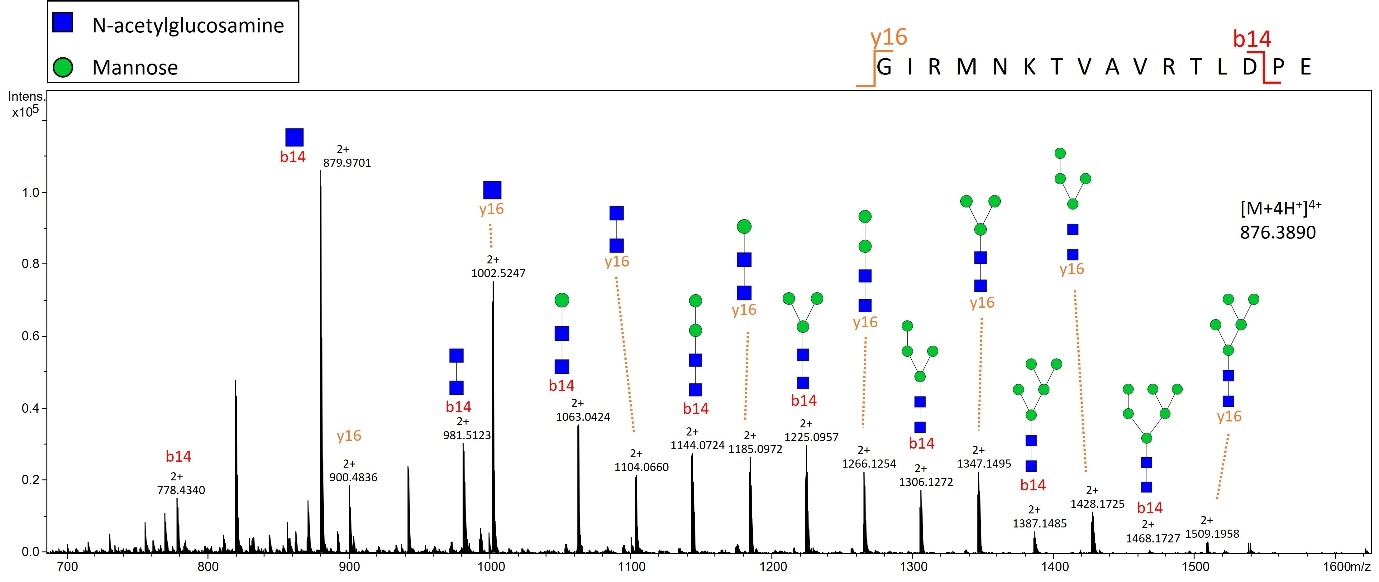
**

**E
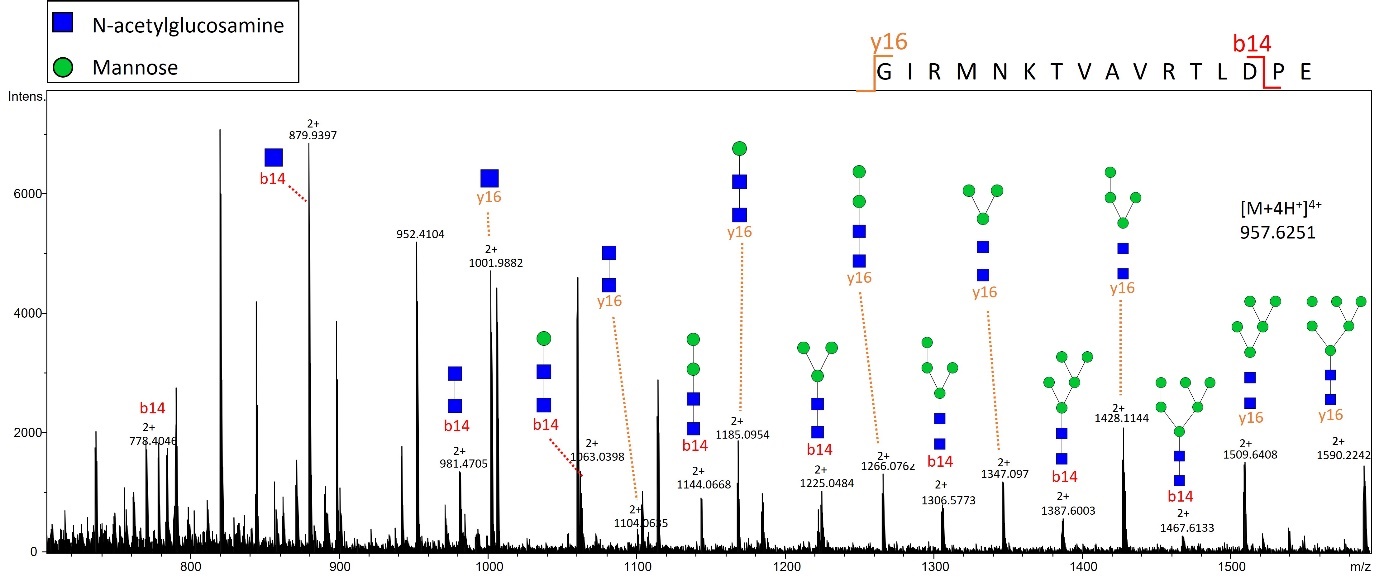
**

**Figure S2** Representative MS/MS fragmentation spectrum. **a,** glycoform C3.Asn939-N2H9. **b**, glycoform C3.Asn85-N2H5. **c**, glycoform C3.Asn85-N2H7. **d**, glycoform C3.Asn939-N2H8. **e**, glycoform C3.Asn939-N2H10.

**
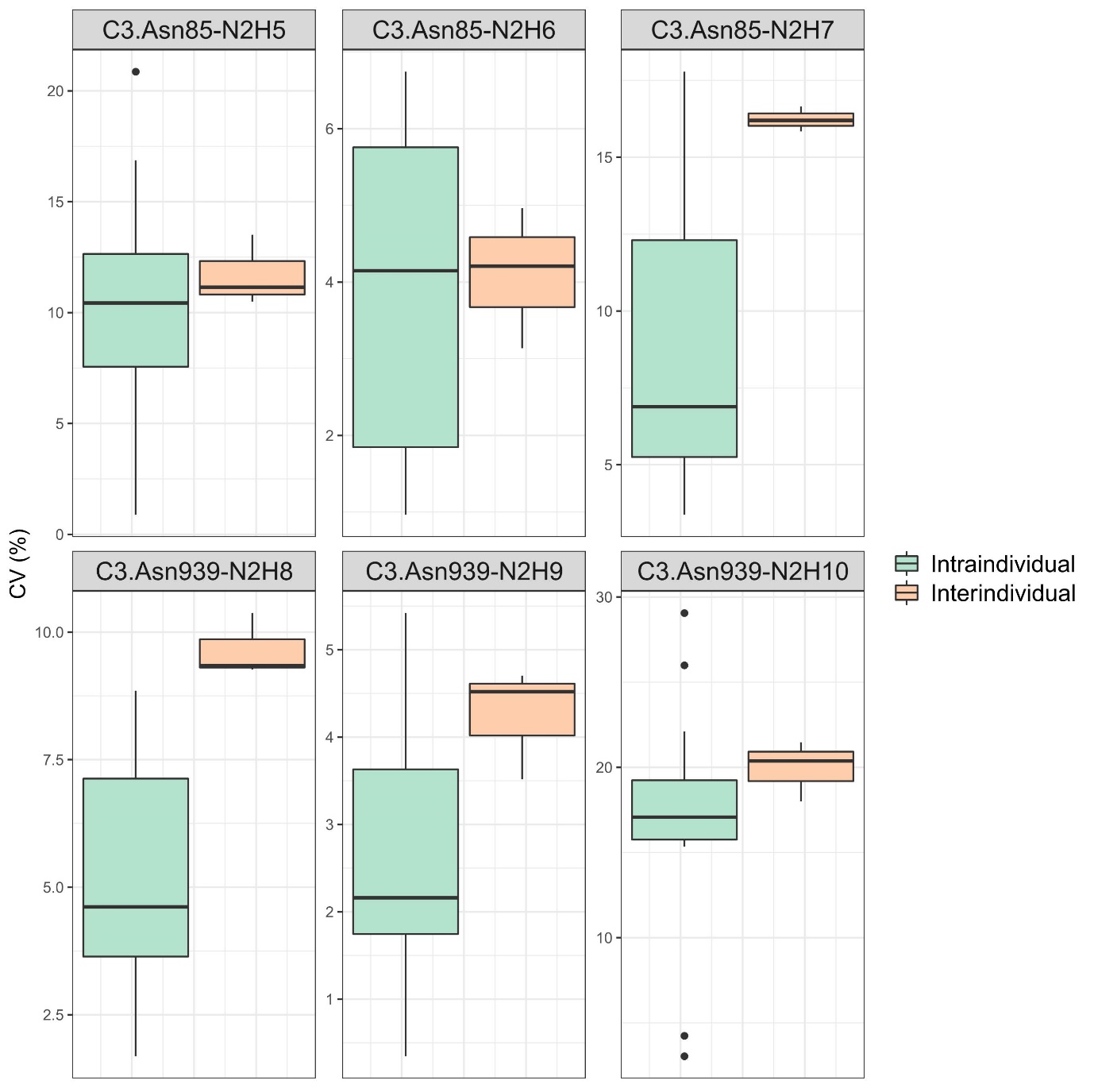
**

**Figure S3** Temporal stability of C3 glycoforms in box plots. Intraindividual CV was measured from longitudinal samples of each subject, while the interindividual CV was computed from all students’ samples within each time point. C3.Asn85 – site Asn 85, C3.Asn939 – site Asn 939, N – N-Acetylglucosamine, H – hexose.
